# Musculoskeletal disorders and psychosocial risks among electricians

**DOI:** 10.1192/j.eurpsy.2024.1711

**Published:** 2024-08-27

**Authors:** I. Sellami, A. Feki, A. Abbes, K. Jmal Hammami, M. Hajjaji, S. Baklouti, M. L. Masmoudi

**Affiliations:** ^1^Occupational medicine, Hedi Chaker Hospital; ^2^ Medecine university; ^3^Rheumatoloy, Hedi Chaker Hospital, Sfax, Tunisia

## Abstract

**Introduction:**

There is a growing concern about the link between musculoskeletal disorders (MSD) and psychosocial risk (PSR) among electricians. Both MSD and PSR represent a threat to the electrician’s health, quality of life and productivity.

**Objectives:**

This study aimed to assess the link between PSR and MSD among electricians.

**Methods:**

The study was conducted in a group from an electricity society. Data were gathered between January-June 2022 using a self-administered questionnaire evaluating socio-professional characteristics, the Nordic musculoskeletal questionnaire during the previous year and the validated French version of the questionnaire KARASEK.

**Results:**

Our study included 68 male electricians. The mean age was 39.2 ± 10.3 years. The average job tenure was 16± 11.4 years. According to the Nordic musculoskeletal questionnaire, 50% of participants experienced pain during the last 12 months.

About half of the electricians had high psychological demand (48.5%), 63.2% had a low latitude, and 76.5% had low social support. According to the Karasek model, tense electricians accounted for 26.5% and assets 22.1%. MSDs were associated with high psychological demand at work (p = 0.02).

**Conclusions:**

This study demonstrated that PSR and MSDs are associated among electricians and are highly prevalent. They represent an important concern of the occupational and safety health system. The prevention of MSD should take into account the specific working conditions of electricians to reduce their exposure to psychosocial risk factors in the workplace.

**Disclosure of Interest:**

None Declared

